# The *Rhizophagus irregularis* Genome Encodes Two CTR Copper Transporters That Mediate Cu Import Into the Cytosol and a CTR-Like Protein Likely Involved in Copper Tolerance

**DOI:** 10.3389/fpls.2019.00604

**Published:** 2019-05-16

**Authors:** Tamara Gómez-Gallego, Karim Benabdellah, Miguel A. Merlos, Ana M. Jiménez-Jiménez, Carine Alcon, Pierre Berthomieu, Nuria Ferrol

**Affiliations:** ^1^Departamento de Microbiología del Suelo y Sistemas Simbióticos, Estación Experimental del Zaidín, Consejo Superior de Investigaciones Científicas, Granada, Spain; ^2^Genomic Medicine Department, GENYO, Centre for Genomics and Oncological Research, Pfizer-University of Granada-Andalusian Regional Government, Granada, Spain; ^3^Biochimie et Physiologie Moléculaire des Plantes, Institut National de la Recherche Agronomique, Centre National de la Recherche Scientifique, Université de Montpellier, Montpellier SupAgro, Montpellier, France

**Keywords:** arbuscular mycorrhizal fungi, copper homeostasis, copper transporters, CTR family, *Rhizophagus irregularis*, symbiosis

## Abstract

Arbuscular mycorrhizal fungi increase fitness of their host plants under Cu deficient and toxic conditions. In this study, we have characterized two Cu transporters of the CTR family (RiCTR1 and RiCTR2) and a CTR-like protein (RiCTR3A) of *Rhizophagus irregularis*. Functional analyses in yeast revealed that *RiCTR1* encodes a plasma membrane Cu transporter, *RiCTR2* a vacuolar Cu transporter and *RiCTR3A* a plasma membrane protein involved in Cu tolerance. *RiCTR1* was more highly expressed in the extraradical mycelia (ERM) and *RiCTR2* in the intraradical mycelia (IRM). In the ERM, *RiCTR1* expression was up-regulated by Cu deficiency and down-regulated by Cu toxicity. *RiCTR2* expression increased only in the ERM grown under severe Cu-deficient conditions. These data suggest that RiCTR1 is involved in Cu uptake by the ERM and RiCTR2 in mobilization of vacuolar Cu stores. Cu deficiency decreased mycorrhizal colonization and arbuscule frequency, but increased *RiCTR1* and *RiCTR2* expression in the IRM, which suggest that the IRM has a high Cu demand. The two alternatively spliced products of *RiCTR3, RiCTR3A* and *RiCTR3B*, were more highly expressed in the ERM. Up-regulation of *RiCTR3A* by Cu toxicity and the yeast complementation assays suggest that RiCTR3A might function as a Cu receptor involved in Cu tolerance.

## Introduction

The transition metal copper (Cu) is a micronutrient acting as a redox active cofactor of key enzymes involved in a wide array of biochemical processes essential for life, such as respiration, superoxide scavenging and iron mobilization (Linder, 1991; Festa and Thiele, 2011). However, when in excess, it becomes toxic due to its ability to displace other metal ions in structural or catalytic protein motifs (Macomber and Imlay, 2009) and through the generation of hydroxyl radicals by Fenton-like reactions (Halliwell and Gutteridge, 1984). Due to the dual nature of Cu, organisms have developed sophisticated homeostatic networks to tightly regulate its intracellular levels, in which membrane transporters mediating Cu uptake and efflux and specific chaperones that handle and deliver Cu to its specific target enzymes play a key role (Puig and Thiele, 2002; Kim et al., 2008; Smith et al., 2017).

In eukaryotes, the major entrance of Cu into the cells occurs through members of the Cu transporter (CTR) family, small integral membrane proteins of variable length (from 150 to 500 amino acid residues) that have three transmembrane (TM) domains and the characteristic signature MetXXXMet-X_12_-GlyXXXGly embedded within TM2 and TM3 (Dumay et al., 2006; De Feo et al., 2007). CTRs are present in the membranes as a homo-oligomer or hetero-oligomer complex, being the cooperation between the different subunits crucial for Cu transport (Puig et al., 2002; Beaudoin et al., 2011). The MetXXXMet motif is located in TM2 and together with a cluster of Met residues in the N terminal domain is involved in Cu sensing and uptake (Puig et al., 2002; Guo et al., 2004), while TM3 harbors the GlyXXXGly motif that is critical for protein folding and oligomerization (Aller et al., 2004). The carboxy terminal domain usually contains Cys and/or His motifs that bind and transfer Cu to cytosolic chaperones for its final targeted distribution (Dancis et al., 1994b; Xiao et al., 2004; Puig, 2014). Additionally, under Cu toxicity this domain allows protein inactivation through conformational structural changes (Wu et al., 2009). This family of transporters has been widely studied in *Saccharomyces cerevisiae*, which encodes three members (Ctr1, Ctr2, and Ctr3). Ctr1 and Ctr3 are functionally redundant plasma membrane transporters that mediate Cu acquisition from the environment (Dancis et al., 1994a; Peña et al., 2000), while Ctr2 is located in the vacuolar membrane and pumps vacuolar Cu stores to the cytosol (Portnoy et al., 2001; Rees et al., 2004). *Ctr1* and *Ctr3* expression is highly induced under Cu deficiency in order to facilitate high-affinity Cu acquisition and Ctr2 mobilizes Cu vacuolar stores when Cu levels are extremely low. Apart from other yeasts (Bellemare et al., 2002; Marvin et al., 2003; Beaudoin et al., 2011), CTRs have been characterized in the basidiomycetes *Pleurotus ostreatus* (Penas et al., 2005), *Coprinopsis cinerea* (Nakagawa et al., 2010) and *Amanita strobiliformis* (Beneš et al., 2016), as well as in the filamentous ascomycetes *Podospora anserina* (Borghouts et al., 2002), *Colletotrichum gloeosporioides* (Barhoom et al., 2008) and *Neurospora crassa* (Korripally et al., 2010). Fungal Ctr proteins have been shown to be involved in different processes. For example, the vacuolar Cu transporter Ctr2 of the plant pathogen *C. gloeosporioides* is essential for optimal spore germination and pathogenesis (Barhoom et al., 2008) and the high-affinity Cu transporter TCU-1 of *N. crassa* is essential for saprophytic conidical germination and vegetative growth under Cu limiting conditions (Korripally et al., 2010). However, very little is known about the mechanisms of Cu uptake in arbuscular mycorrhizal (AM) fungi, the most ancient and widespread fungal plant symbionts.

Arbuscular mycorrhizal fungi are soil-borne microorganisms of the subphylum Glomeromycotina within the Mucoromycota (Spatafora et al., 2016) that establish a mutualistic symbiosis with the majority of land plants. In this mutualistic relationship the fungal partner receives carbon compounds from the plant in exchange of low mobility mineral nutrients in soil, mainly phosphorus and some micronutrients, such as Zn and Cu (Smith and Read, 2008; Lanfranco et al., 2018). Besides improving plant mineral nutrition, AM fungi increase plant ability to overcome biotic and abiotic stress conditions, such as salinity, drought and metal toxicity (Ruiz-Lozano, 2003; Pozo et al., 2013; Ferrol et al., 2016). It is noteworthy the ability of AM fungi to increase plant fitness under deficient and excess Cu availability (Lehmann and Rillig, 2015; Ferrol et al., 2016). As revealed by isotopic labeling experiments, improvements in Cu nutrition by AM fungi are due to the capability of the extraradical mycelia (ERM) to absorb the micronutrient beyond the depletion zone that develops around the roots (Li et al., 1991; Lee and George, 2005). On the other hand, increased plant performance in Cu-polluted soils is mainly due to the ability of the fungus to act as a barrier for Cu entry into the plant tissues (Ferrol et al., 2016; Merlos et al., 2016). Despite the central role Cu transporters play in all organisms to cope with a range of Cu availability, from scarcity to excess, the mechanisms of Cu import in AM fungi have not been characterized yet. In a previous genome-wide analysis of metal transporters in the AM fungus *Rhizophagus irregularis*, we identified three genes putatively encoding Cu transporters of the CTR family that mediate Cu transport into the cytosol (Tamayo et al., 2014). With the aim to get further insights into the mechanisms of Cu homeostasis in AM fungi, in this work we carried out the first characterization of the *R. irregularis* CTR transporters.

## Materials and Methods

### Biological Materials and Growth Conditions

The AM fungal isolate used in this study was *Rhizophagus irregularis* (Blaszk., Wubet, Renker & Buscot) C. Walker & A. Schüßler DAOM 197198. The fungal inoculum used for the root organ cultures and for the seedlings was obtained in monoxenic cultures. AM monoxenic cultures were established according to St-Arnaud et al. (1996), with some modifications. Briefly, Ri T-DNA transformed carrot (*Daucus carota* L. clone DC2) roots were cultured with *R. irregularis* in solid M medium (Chabot et al., 1992) in two-compartment Petri dishes. Cultures were started in one compartment by placing the fungal inoculum (ERM, spores and mycorrhizal roots fragments) and some pieces of carrot roots. Plates were incubated in the dark at 24°C for 6–8 weeks until the other compartment of the Petri dish was profusely colonized by the fungus and roots (root compartment). The older compartment was removed and filled with liquid M medium without sucrose (M-C medium) and the fungal mycelium was allowed to colonize this compartment (hyphal compartment) during the two subsequent weeks (Control plates).

For the Cu deficiency treatments, monoxenic cultures were established in media without Cu and started with roots and inoculum previously grown either in M media, which contains 0.5 μM CuSO_4_, (moderate Cu deficiency treatment) or in M media without Cu (severe Cu deficiency treatment), and grown in the same conditions than the control plates but in media lacking Cu. ERM and mycorrhizal roots grown, respectively, in the hyphal and root compartment of each plate were collected, rapidly dried on filter paper, immediately frozen in liquid N and stored at -80°C until used. An aliquot of the roots from each treatment was separated to estimate mycorrhizal colonization.

For the Cu toxicity and H_2_O_2_ treatments, the M-C medium of the hyphal compartment was removed and replaced with fresh liquid M-C medium (Control, 0.5 μM CuSO_4_) or with M-C medium supplemented with 250 μM CuSO_4_, 500 μM CuSO_4_ or 1 mM H_2_O_2_ and incubated at 24°C. The time of medium exchange was referred as time 0. Mycelia were collected 1, 2, and 7 days after Cu addition and 1 h after H_2_O_2_ supplementation.

For gene expression comparison between ERM and IRM, several non-mycorrhizal carrot roots pieces were placed on the top of a densely fungal colonized compartment and grown for 15 days at 24°C. Roots were carefully collected with tweezers under a bionocular microscope trying to remove the attached extraradical hyphae, frozen in liquid N and stored at -80°C until used. An aliquot of root fragments was separated to estimate mycorrhizal colonization.

*Rhizophagus irregularis* ERM was also collected from mycorrhizal plants grown in the *in vivo* whole plant bidimensional experimental system described by Pepe et al. (2017) with some modifications (Supplementary Figure S1). Briefly, chicory (*Cichorium intybus* L.) seeds were surface-sterilized and germinated for 10–15 days in sterilized sand. Seedlings were transplanted into 50 mL pots filled with sterilized sand and inoculated with spores, ERM and colonized roots obtained from monoxenic cultures. Pots were placed in sun-transparent bags (Sigma-Aldrich, B7026) and maintained during 1 month in a growth chamber at 24°C/21°C day/night and 16 h light photoperiod. The root system of each plant was cleaned, wrapped in a nylon net (41 μM mesh, Millipore NY4100010) and placed between two 13 cm membranes of mixed cellulose esters (0.45 μm pore diameter size, MF-Millipore HAWP14250) in 14 cm diameter Petri dishes having a hole on the edge to allow plant shoot growth and containing sterilized sand. Petri plates containing plants were sealed with parafilm, wrapped with aluminum foil, placed into sun-transparent bags and maintained in a growth chamber. Plants were watered weekly with a 0.5X modified Hoagland nutrient solution containing 125 μM KH_2_PO_4_ and 0.16 μM CuSO_4_ (control treatment) or without Cu (Cu deficiency treatment). Each treatment consisted of five replicates. Petri dishes were opened 2 weeks after preparing the root sandwiches and ERM spreading from the nylon net onto the membranes was collected with tweezers, frozen in liquid N and stored at -80°C until used. Roots wrapped in the nylon net were also frozen and stored at -80°C. An aliquot of the roots was separated to estimate mycorrhizal colonization.

The *Saccharomyces cerevisiae* strains used in this study were MPY17 (*ctr1Δctr3Δ*), a double-mutant lacking the plasma membrane transporters Ctr1 and Ctr3 (Peña et al., 1998) and MPY17 *ctr2Δ* (*ctr1Δctr2Δctr3Δ*), a triple mutant lacking also the vacuolar transporter ScCtr2 (Rees et al., 2004) and WYT (*yap1Δ*) a strain lacking the transcription factor yap1 (Kuge and Jones, 1994). Detailed characteristics of yeast strains are listed in Supplementary Table S1. Yeast cells were maintained on YPD or minimal synthetic dextrose (SD) medium, supplemented with appropriate amino acids.

### Mycorrhizal Colonization

Histochemical quantification of mycorrhizal colonization was performed according to Trouvelot et al. (1986) using the Mycocalc program^1^ in root samples previously cleared with 10% KOH and stained with 0.05% trypan blue (Phillips and Hayman, 1970). The abundance of the AM fungus was also assessed by determining the expression level of the *R. irregularis* elongation factor 1α (*RiEF1α*; GenBank Accession No. DQ282611), using as internal control the elongation factor 1α of the corresponding host plant (*Daucus carota* L. *DcEF1α*, GenBank Accession No. XM_017391845; *Cichorium intybus* L. *CiEF1α*, GenBank Accession No. KP752079).

### Nucleic Acids Extraction and cDNA Synthesis

*Rhizophagus irregularis* genomic DNA was isolated from ERM developed in the hyphal compartment of control plates using the DNeasy Plant Mini Kit (Qiagen), according to the manufacturer’s instructions.

Total RNA extraction from fungal ERM and mycorrhizal carrot roots developed in monoxenic cultures was performed using the Plant RNeasy Kit (Qiagen) following manufacturer’s instructions. Total RNA from mycorrhizal chicory roots was isolated using the phenol/SDS method followed by LiCl precipitation (Kay et al., 1987). RNAs were DNase treated with the RNA-free DNase set (Qiagen) according to manufacturer’s instructions and quantified using the NanoDrop 1000 Spectrophotometer (Thermo Scientific). cDNAs were synthetized from 1 μg of total DNase-treated RNAs in a 20 μL reaction containing 200 U of SuperScript III Reverse Transcriptase (Invitrogen) and 2.5 μM oligo (dT)_20_ primer (Invitrogen), following the manufacturer’s instructions.

### RiCTRs Identification and Sequences Analyses

Candidate gene sequences putatively encoding RiCTRs were previously identified by Tamayo et al. (2014). Additional Blastp searches were performed in the filtered model datasets of the *R. irregularis* isolates DAOM197198 v2.0 and A1, A4, A5, B3, and C2 v1.0 (Chen et al., 2018) recently deposited at the JGI website^2^, using as a query the previously identified RiCTR candidates. These sequences were also used to identify CTR homologs via Blastp in the sequence datasets of other Glomeromycotina species deposited on the JGI (*Gigaspora rosea* v1.0, *Rhizophagus cerebriforme* DAOM 227022 v1.0 and *Rhizophagus diaphanus* v1.0 (Morin et al., 2019) and NCBI [*Diversispora epigaea* (Sun et al., 2019) and *Rhizophagus clarus* (Kobayashi et al., 2018)] websites.

Sequence analyses were performed using the DNAstar Lasergene software package (DNAstar), BLAST tool of NCBI^3^ and Clustal Omega for sequence alignments^4^. Gene promoter sequences were screened for the presence of regulatory *cis* elements employing the tools included in the Promoter Database of *Saccharomyces cerevisiae* SCPD^5^. Specific Cu responsive elements (CuREs) and AP-1 sites were further screened through DNA pattern matching analyses in the fungal RSAT server^6^. Identity and Similarity percentages between proteins were calculated using Ident and Sim tool from Sequence Manipulation Suite^7^. Conserved domains of proteins were identified using the Conserved Domain Database at NCBI^8^, predictions of putative TM domains via the TMHMM Server v.2.0^9^, the SOSUI engine v. 1.11^10^ and the TOPCONS web server^11^, Structural models of the RiCTRs were generated using MyDomains tool of Prosite^12^. Phylogenetic analyses were performed via the Neighbor-Joining method implemented in the Molecular Evolutionary Genetics Analysis software v. 6. (MEGA), with 1,000 bootstrap replicates, using Poisson model and pairwise deletion of gaps options for distance computation.

### Gene Isolation

The cDNA sequences of the 5′ and 3′ends of *RiCTR1-3* were confirmed and completed, when necessary, by RACE using the SMARTer^®^ RACE 5′/3′ kit (Clontech) according to the manufacturer’s protocol. The primers used for RACE reactions are listed in Supplementary Table S2. Genomic clones and full length cDNAs were obtained by PCR amplification of *R. irregularis* genomic DNA and cDNA, respectively, from ERM grown under control conditions in monoxenic cultures, using a set of primers flanking the complete open reading frames (Supplementary Table S2). PCR products were cloned into the pGEM-T Easy vector (Promega), following manufacturer’s instructions. Plasmids were amplified by transformation of chemically *Escherichia coli* DH5α competent cells according to standard procedures and purified using the GenElute^TM^ Plasmid Miniprep Kit (Sigma-Aldrich). All plasmids were checked by sequencing (ABI PRISM 3130*xl* Genetic Analyzer, Applied Biosystems, Carlsbad, CA, United States).

### Functional Complementation Analyses in Yeast

RiCTR open reading frames were sub-cloned between *Sma*I and *Xho*I sites (*RiCTR1*) or *Pst*I and *Sal*I sites (*RiCTR2* and *RiCTR3*) of the yeast expression vector pDR196. For this purpose, the respective full-length cDNA sequences were flanked with the sequences recognized by the corresponding restriction enzymes by PCR using the primers described in Supplementary Table S2. PCR products were cloned into the pGEM-T Easy vector (Promega), following manufacturer’s instructions. The full-length cDNAs were then isolated from the pGEM-T Easy vector by digestion with the corresponding restriction enzymes and ligated into the digested pDR196 vector. All constructs were verified by sequencing. The *S. cerevisiae* strains *ctr1Δctr3Δ, ctr1Δctr2Δctr3Δ* and *yap-1Δ* were transformed with the corresponding pDR196-*RiCTR* constructs or with the empty vector using a lithium acetate-based method (Gietz and Schiestl, 2007). Transformants were selected in SD medium by autotrophy to uracil. For drop tests, transformants were grown to exponential phase in SD medium without uracil. Cells were harvested by centrifugation, washed twice and adjusted to a final OD_600_ of 1. Then, 5 μL of serial 1:10 dilutions were spotted on the corresponding selective medium. The transformed *ctr1Δctr3Δ* and *ctr1Δctr2Δctr3Δ* strains were spotted onto a non-fermentable carbon source ethanol-glycerol medium (YPEG: 1% yeast extract, 2% bacto-peptone, 2% ethanol, 3% glycerol, 1.5% bacto-agar) supplemented with 0, 10, or 20μM CuSO_4_. The transformed *yap-1Δ* cells were spotted onto SD without uracil supplemented either with 1.5 mM CuSO_4_ or 0.5 mM H_2_O_2._

### Protein Localization

Subcellular localization of RiCTR1-3 was assessed with N or C terminal fusions of these genes to the enhanced green fluorescent protein (eGFP) in the *S. cerevisiae* triple mutant *ctr1Δctr2Δctr3Δ* or in *yap-1Δ*. The coding sequences of *RiCTR1, RiCTR2* and *RiCTR3A* were cloned with or without their stop codon into pENTR/D-TOPO (Invitrogen) via TOPO reactions and then into the destination vectors pFGWDR196 or pGWFDR196 by using the Gateway LR Clonase recombination system (Invitrogen) for eGFP-tagging at the amino- or carboxy-terminus, respectively. Primers pairs used are listed in Supplementary Table S2. The corresponding yeast mutants were transformed with the resulting pFGWDR/pGWFDR196-*RiCTR* constructs or with the empty vector (negative control). Functionality of the GFP-tagged versions of RiCTRT1, RiCTR2, and RiCTR3A was tested in complementation assays, as previously described. For the protein localization assays, yeast cells were grown to exponential phase in liquid SD without uracil and visualized using a Leica TCS SP8 laser scanning microscope with a 63× oil N.A. 1.4 immersion objective. Emission fluorescence of GFP was excited at 488 nm and the emitted signal was collected between 500 and 540 nm. To reduce overexpression artifacts, yeast cells were treated just before visualization with the protein synthesis inhibitor cycloheximide (100 μM) for 45 min. Images were processed using ImageJ software.

### Gene Expression Analyses

Gene expressions were analyzed by real-time quantitative RT-PCR using an iQ^TM^ 5 Multicolor Real-Time PCR Detection System (Bio-Rad). Each 20 μl reaction contained 1 μl of a 1:10 dilution of the cDNA, 200 nM each primer and 10 μl iQ^TM^ SYBR Green Supermix (Bio-Rad). The primer sets used are listed in Supplementary Table S2. The program consisted in an initial incubation at 95°C for 3 min, followed by 38 cycles of 95°C for 30 s, 58°C for 30 s and 72°C for 30 s, where the fluorescence signal was measured. The specificity of the PCR amplification procedure was checked with a heat-dissociation protocol (from 58 to 95°C) after the final PCR cycle. Since RNA extracted from mycorrhizal roots contains plant and fungal RNAs, specificity of the primers pairs was also analyzed by PCR amplification of carrot and chicory genomic DNA and cDNA from non-mycorrhizal carrot and chicory roots. Specificity of the *RiCTR3A* and *RiCTR3B* primer pairs was analyzed by PCR amplification of the *RiCTR3A* and *RiCTR3B* plasmid DNAs. RT-PCR determinations were performed in three independent biological samples with the threshold cycle (Ct) determined in duplicate in at least two independent PCR experiments. The relative abundance of the transcripts was calculated by using 2^-ΔΔCT^ method (Livak and Schmittgen, 2001) and normalized according to the expression of the *R. irregularis* elongation factor 1α (*RiEF1α*; GenBank Accession No. DQ282611).

### Statistical Analyses

IBM SPSS Statistic software v.23 was used for the statistical analysis of the means and standard error determinations. Data were subjected to the Student’s *t*-test when two means were compared or by one-way ANOVA followed by a Fisher’s LSD to find out differences among groups of means (*P* < 0.05). All the analyses are based on at least three biological replicates per each treatment (*n* ≥ 3).

## Results

### Features of the *R. irregularis* CTR Proteins

**FIGURE 1:**
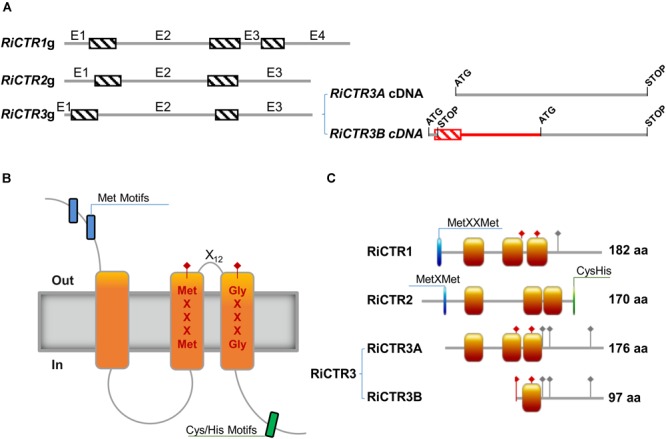
Structure of the *R. irregularis* CTR proteins. **(A)** Exon/intron organization of the *R. irregularis* CTR genes. Exons (E) and introns are represented by gray lines and dashed boxes, respectively. The two transcript variants of *RiCTR3* are illustrated at the right. The coding regions of the two spliced variants of *RiCTR3* are shown in gray and the non-coding in red. The start and stop codons are indicated. **(B)** Typical topological model of a CTR transporter. **(C)** Schematic representation of the structure of the *R. irregularis* CTR transporters. Orange boxes illustrate transmembrane domains, blue boxes Met motifs and green boxes Cys and His motifs. Red diamonds show the positions of the MetXXXMet and the GlyXXXGly motives of the CTRs master signature and gray diamonds the positions of the C-terminal His/Cys residues. Amino acid lengths are also indicated.

The *R. irregularis CTR1, CTR2*, and *CTR3* full-length cDNA sequences were obtained by RACE using gene-specific primers based on the sequences described by Tamayo et al. (2014) [GenBank Accession No. /JGI IDs: PKC06371/1491164 (*RiCTR1*), EXX67481/1726366 (*RiCTR2*) and PKC14368/495436 (*RiCTR3*)]. Interestingly, two types of *CTR3* transcripts were identified in the *R. irregularis* ERM, *RiCTR3A* of 531 bp and *RiCTR3B* of 606 bp. Comparisons of the full-length cDNAs with the genomic sequences revealed the presence of three introns in *RiCTR1* and two in *RiCTR2* and *RiCTR3*, all of them flanked by the canonical splicing sequences GT and AG at the 5′ and 3′ ends, respectively (Figure 1A). Alignment of the *RiCTR3A* and *RiCTR3B* transcripts with the *RiCTR3* gene sequence indicates that both transcripts are alternatively spliced products of the same gene, as the *RiCTR3A* and *RiCTR3B* sequences are contained within the genomic sequence. *RiCTR3B*, the longest *RiCTR3* variant, contains the first intron after the *RiCTR3A* start codon generating a premature termination codon-containing mRNA. However, an additional start codon located within the second exon becomes available to produce a protein that contains the last 97 amino acids of RiCTR3A.

*RiCTR1, RiCTR2*, and *RiCTR3A* encode proteins of 182, 170, and 176 amino acids, respectively, that have three TM domains with the MetXXXMet-X_12_-GlyXXXGly signature embedded within TM2 and TM3, an intracellular loop connecting TM1 and TM2, the N terminus toward the extracellular space and the C terminus facing the cytosol (Figure 1B,C). RiCTR1 and RiCTR2 present a Met motif, MetXXMet in RiCTR1 and MetXMet in RiCTR2, in the N terminal extracytosolic region 29 and 23 amino acids before TM1, respectively. This methionine motif, which is essential for CTR function (Puig et al., 2002), is absent in RiCTR3A. RiCTR2 harbors a Cys/His motif in the carboxy-terminal region facing the cytoplasm, while RiCTR1 has a single His residue and RiCTR3A a Cys and two His residues (Figure 1C and Supplementary Figure S2). Despite the similar structure and sequence amino acid length of RiCTR1, RiCTR2, and RiCTR3A, similarity between their deduced amino acid sequences is lower than 53%, displaying RiCTR1 and RiCTR3A the highest similarity (Supplementary Table S3). *RiCTR3B* encodes a protein of 97 amino acids that harbors the MetXXXMet-X_12_-GlyXXXGly signature of CTR proteins, but has a single TM domain, the MetXXXMet is mislocalized in the N terminal domain and the GlyXXXGly motif is embedded in its only TM domain.

A phylogenetic analysis of fungal CTR transporters revealed that RiCTR1 and RiCTR3 clustered with the *S. cerevisiae* plasma membrane Ctr1/Ctr3-like Cu transporters and RiCTR2 with the *S. cerevisiae* vacuolar Ctr2-like transporters. Within each clade, all Glomeromycotina sequences were grouped together (Supplementary Figure S3).

*In silico* searches for putative regulatory elements in their promoter sequences resulted in the identification of several core elements identical to the Cu response *cis*-element (CuRE) GTAC present in the promoters of Cu-responsive genes (Jamison McDaniels et al., 1999; Kropat et al., 2005) and the consensus sequence of the AP-1 *cis*-acting element (TTATTAA/TTAGTAA) recognized as a conserved motif in the oxidative stress-responsive genes (Toone and Jones, 1999) (Supplementary Figure S4). Interestingly, the 5′-flanking region of *RiCTR3* is especially rich in AP-1 motifs and contains the preferred DNA binding site (TTACTAA) of the transcription factor YAP1 (Toone and Jones, 1999), which is essential for the oxidative stress response in *S. cerevisiae* (Kuge and Jones, 1994).

### *RiCTR1* and *RiCTR2* Encode Functional Cu Transporters

**FIGURE 2:**
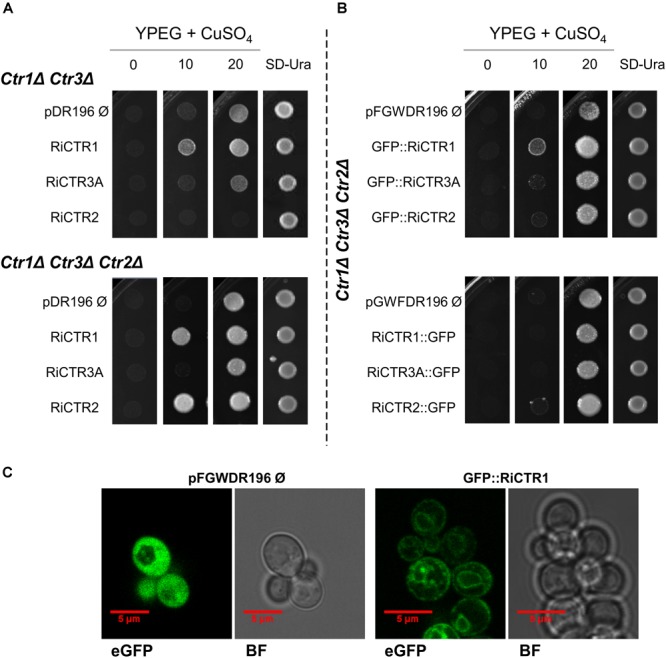
Analysis of the Cu transport activity and subcellular localization of the *R. irregularis* CTRs in yeast. **(A)** *ctr1Δctr3Δ* and *ctr1Δctr2Δctr3Δ* yeast cells transformed with the empty vector or expressing *RiCTR1, RiCTR2, RiCTR3A or RiCTR3B* were plated on YPEG media supplemented with Cu (0, 10, or 20 μM CuSO_4_) or on SD medium without uracil. *ctr1Δctr3Δ* and *ctr1Δctr2Δctr3Δ* plated cells were incubated at 30°C for 4 and 7 days, respectively. RiCTR3A and RiCTR3B had the same effect (see Supplementary Figure S5), only the result with RiCTR3A is illustrated. **(B)** *ctr1Δctr2Δctr3Δ* yeast cells expressing *GFP* (empty vector), N-terminal (upper panel) or C-terminal (lower panel) *GFP*-tagged versions of *RiCTR1, RiCTR2* and *RiCTR3A* were plated on YPEG media supplemented with Cu (0, 10, or 20 μM CuSO_4_) or on SD medium without uracil. Plates were incubated at 30°C for 7 days. **(C)** *ctr1Δctr2Δctr3Δ* cells expressing *GFP* (the empty vector pFGWDR196) and *GFP::RiCTR1* were visualized with a confocal microscope. eGFP, enhanced GFP fluorescence; BF, bright field.

Since AM fungi cannot be genetically manipulated, functionality of the RiCTRs was assessed in yeast by testing their ability to revert the inability of the double (*ctr1Δctr3Δ*) and triple (*ctr1Δctr2Δctr3Δ*) *S. cerevisiae* CTR mutants, which lack the plasma membrane Ctr1 and Ctr3 Cu transporters and the plasma membrane Ctr1/Ctr3 and the vacuolar Ctr2 transporters, to grow on a non-fermentable carbon source at low Cu concentrations. This growth defect is due to the inability of the cytochrome c oxidase to obtain its Cu cofactor, resulting in a defective mitochondrial respiratory chain (Dancis et al., 1994a; Glerum et al., 1996; Rees et al., 2004). To perform the yeast complementation assays, the full-length cDNA coding sequences of *RiCTR1, RiCTR2, RiCTR3A*, or *RiCTR3B* were expressed under the control of the yeast PMA1 promoter in both yeast CTR mutants and plated on ethanol-glycerol (YPEG) medium supplemented with different Cu concentrations. The empty vector-expressing cells were unable to grow on YPEG medium containing <20 μM Cu (Figure 2A). Expression of *RiCTR1* restored the inability of the *ctr1Δctr3Δ* and *ctr1Δctr2Δctr3Δ* yeast strains to grow on YPEG medium supplemented with 10 μM Cu, indicating that RiCTR1 is a functional homolog of the yeast plasma membrane Cu transporters Ctr1/Ctr3. RiCTR2 complemented the inability of the *ctr1Δctr2Δctr3Δ* mutant strain to grow on YPEG medium supplemented with 10 μM Cu but not of the double mutant lacking the two plasma membrane transporters, which suggests that RiCTR2 is a functional homolog of the *S. cerevisiae* vacuolar transporter Ctr2. However, none of the *RiCTR3* variants rescued the phenotype of either the double or the triple CTR mutants (Figure 2A and Supplementary Figure S5), which was expected since their encoded proteins did not contain the required features for CTR function.

Subcellular localization of RiCTR1 and RiCTR2 was assessed in the heterologous system by expressing N- and C-terminal GFP-tagged versions of these proteins in the *ctr1Δctr2Δctr3Δ* strain and visualizing the fusion proteins with a confocal fluorescence microscope. *S. cerevisiae* cells transformed with the empty vector and expressing GFP under the control of the PMA1 promoter were used as a negative control; and functionality of the RiCTR1-2 fusion proteins was assessed before their visualization (Figure 2B). The control cells expressing the soluble GFP showed a general cytosolic fluorescence (Figure 2C). The RiCTR1-GFP, RiCTR2-GFP and GFP-RiCTR2 fusion proteins were unable to revert the mutant phenotype of the *ctr1Δctr2Δctr3Δ* strain and were expressed within the perinuclear endoplasmic reticulum region (data not shown), indicating that the fusion proteins failed to exit the endoplasmic reticulum. As expected from the complementation assays, the *GFP-RiCTR1*-expressing cells showed a clear fluorescent signal at the cell periphery indicative of a plasma membrane localization. GFP-RiCTR1 was also localized within the perinuclear endoplasmic reticulum membrane, a phenomenon commonly found in yeast membrane protein overexpression assays (Figure 2C).

### *RiCTR* Genes Are Differentially Expressed in the IRM and ERM

**FIGURE 3:**
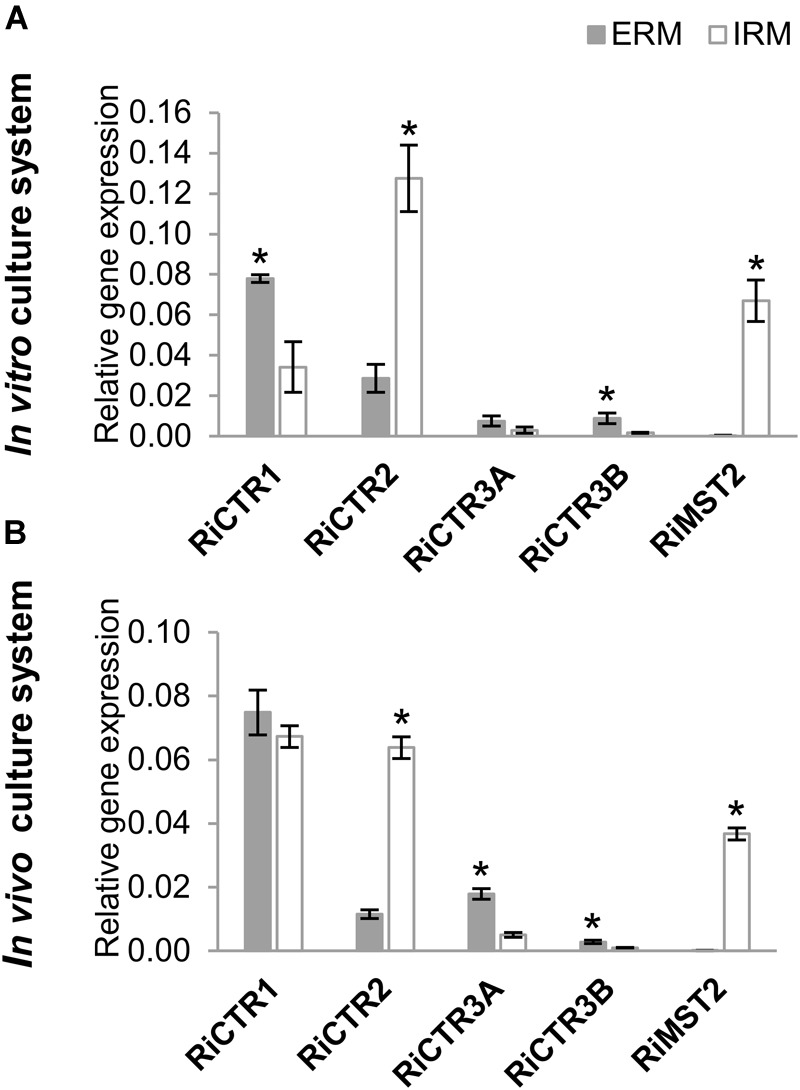
*RiCTR*s expression levels in the *R. irregularis* ERM and IRM. **(A)** *R. irregularis* ERM and mycorrhizal carrot roots (IRM) were grown in compartmented monoxenic cultures (*in vitro* culture system). **(B)** *R. irregularis* ERM and mycorrhizal chicory roots (IRM) were grown in the whole plant bidimensional experimental system (*in vivo* culture system). Relative gene expression levels were calculated by the 2^-ΔCT^ method using *RiEF1α* as a normalizer. Bars represent standard error. Asterisks show statistically significant differences (*P* < 0.05; *n* = 4) between ERM and IRM.

To gain information about the expression of *RiCTR1-3* during symbiosis and about their relative abundance in the intraradical mycelia (IRM) and ERM, their expression levels were determined by real time quantitative RT-PCR (RT-qPCR) in ERM grown in liquid monoxenic cultures and in the *in vivo* sandwich system, and in the IRM developed in carrot roots grown *in vitro* for 2 weeks on a densely colonized hyphal compartment and devoid of ERM and in mycorrhizal chicory roots collected from the *in vivo* sandwich system. Mycorrhizal colonization levels of the carrot and chicory roots were 10 and 78%, respectively. As a reference for fungal activity, we measured transcript levels of the *R. irregularis* high-affinity monosaccharide transporter *RiMST2*, which is highly expressed in the IRM during AM symbiosis (Helber et al., 2011). In both experimental systems, *RiCTR1* was the isoform more highly expressed in the ERM and the expression levels of *RiCTR2* were higher in the IRM than in the ERM. Expression levels of the two spliced-variants of *RiCTR3* were very low in both fungal structures and more highly expressed in the ERM (Figure 3).

### Cu Deficiency Inhibits AM Colonization and Regulates *RiCTR* Expression in the IRM

**FIGURE 4:**
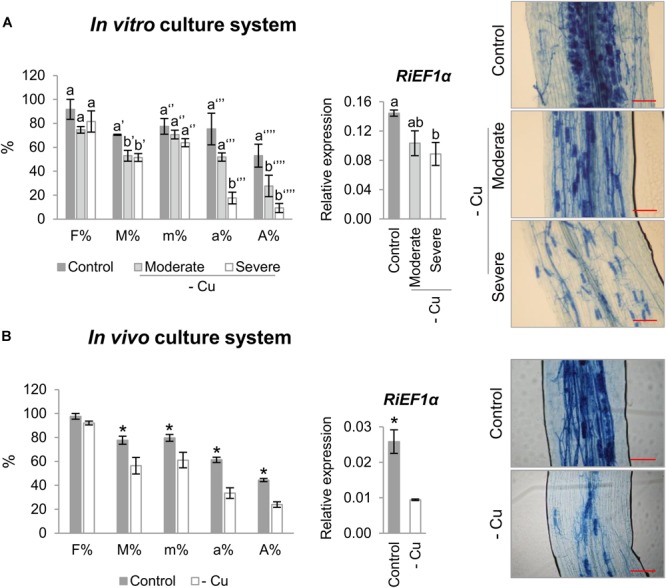
Effect of Cu deficiency on mycorrhizal colonization. **(A)** Mycorrhizal colonization of carrot roots grown in monoxenic cultures in M media (control, 0.5 μM Cu) or in M media lacking Cu in plates started either with roots and inoculum previously grown in M media containing 0.5 μM CuSO_4_ (moderate Cu deficiency) or without Cu (severe Cu deficiency). **(B)** Mycorrhizal colonization of chicory roots grown in the whole plant bidimensional experimental system fertilized with half-strength Hoagland solution (control, 0.16 μM Cu) or with a modified nutrient solution without Cu. Colonization rates were determined by using the Trouvelot method after histochemical staining and by determining the expression level of the *R. irregularis* elongation factor 1α (*RiEF1α*). The relative expression of Ri*EF1α* was calculated using the 2^-ΔCT^ method with *EF1α* of the corresponding host plant as internal control. Bars represent standard error. Different letters indicate significant differences (*P* < 0.05; *n* = 4) between treatments and asterisks statistically significant differences (*P* < 0.05; *n* = 4) in comparison with the control. F%, frequency of mycorrhiza in the root system; M%, intensity of the mycorrhizal colonization in the root system; m%, intensity of the mycorrhizal colonization in the root fragments; a%, arbuscule abundance in mycorrhizal parts of root fragments; A%, arbuscule abundance in the root system. Scale bar of the left panels: 100 μm.

To further understand the role of RiCTR1-3 in the intraradical phase of the fungus, their expression levels were analyzed in mycorrhizal carrot roots grown *in vitro* in monoxenic cultures and in mycorrhizal chicory roots grown *in vivo* in the sandwich system under Cu-optimal and -deficient conditions. Interestingly, irrespective of the culture method, development of the roots under Cu-deficient conditions decreased mycorrhizal intensity and arbuscule frequency (Figure 4). These results were confirmed by determining the transcript levels of the *R. irregularis* elongation factor *RiEF1α* by qRT-PCR (Figure 4).

**FIGURE 5:**
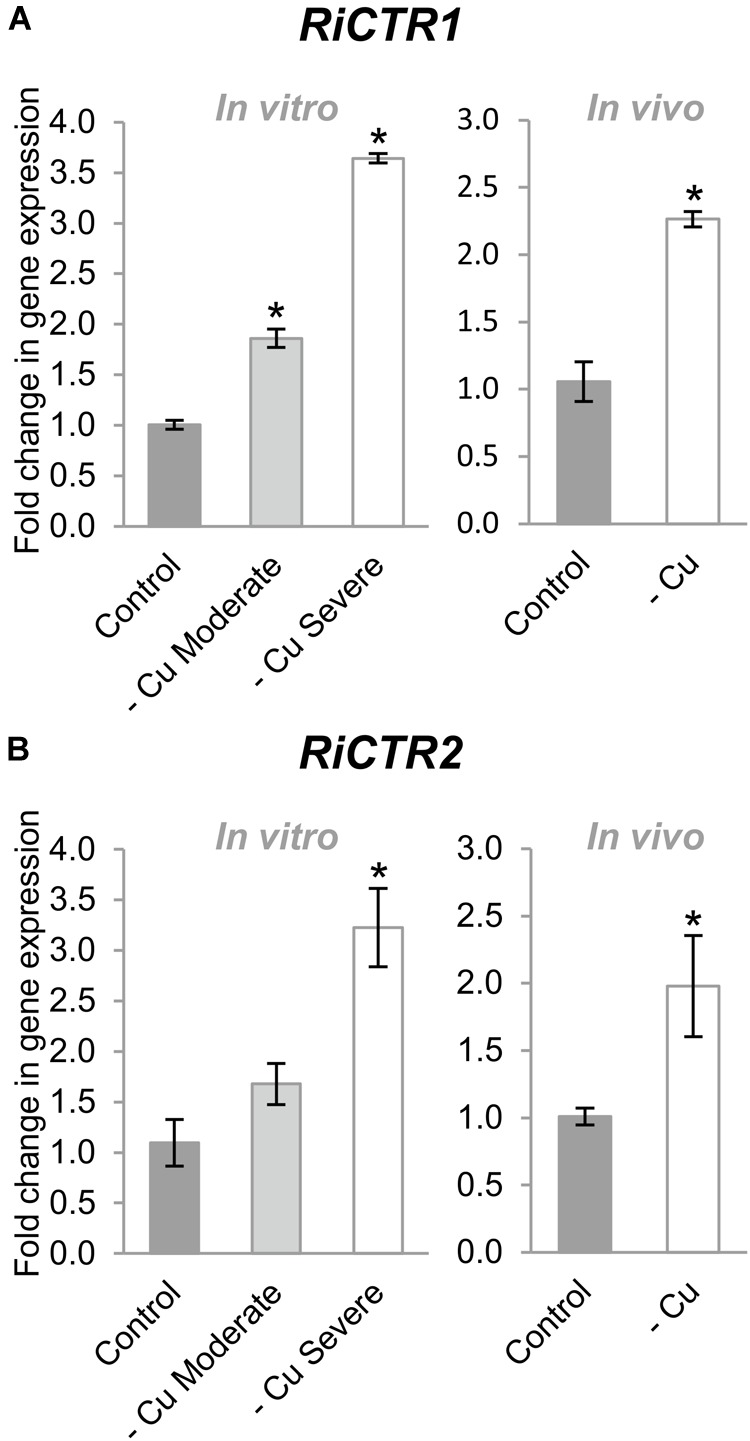
Effect of Cu deficiency on *RiCTR1* and *RiCTR2* IRM expression. **(A)** *RiCTR1* and **(B)** *RiCTR2* expression in mycorrhizal carrot roots developed in monoxenic cultures in M media (control, 0.5 μM Cu) or in M media lacking Cu in plates started either with roots and inoculum previously grown in M media containing 0.5 μM CuSO_4_ (moderate Cu deficiency) or without Cu (severe Cu deficiency) (left panel) and in mycorrhizal chicory roots grown in the whole plant bidimensional experimental system and fertilized with half-strength Hoagland solution (control, 0.16 μM Cu) or with a modified nutrient solution without Cu (right panel). Relative gene expression levels were calculated by the 2^-ΔΔCT^ method using *RiEF1α* as a normalizer. Bars represent standard error. Asterisks show statistically significant differences (*P* < 0.05; *n* = 4) in comparison to the corresponding control value.

Cu deficiency increased *RiCTR1* expression in the IRM developed in the carrot root organ cultures and in the chicory roots grown in the sandwich system (Figure 5A). However, *RiCTR*2 expression was only up-regulated in the IRM of the carrot roots grown under the severe Cu deficiency treatment and of the chicory roots fed with a nutrient solution without Cu (Figure 5B). None of the *RiCTR3* splicing variants were detected in the mycorrhizal roots grown under Cu-limiting conditions, probably because their low expression levels in the IRM and the decrease in mycorrhizal colonization.

### *RiCTRs* Expression in the ERM Is Regulated by Cu Availability

To get further insights into the role of the *R. irregularis* CTR family members on fungal Cu homeostasis, their gene expression patterns were assessed in ERM grown monoxenically under Cu deficient and toxic conditions. Given that development of the ERM was seriously inhibited when the hyphal compartment of the split Petri dishes was supplied with high Cu levels (data not shown), the Cu toxicity treatments were applied by exposing the ERM grown in M media to 250 μM CuSO_4_ for 1, 2, and 7 days or to 500 μM CuSO_4_ for 1 and 2 days.

**FIGURE 6:**
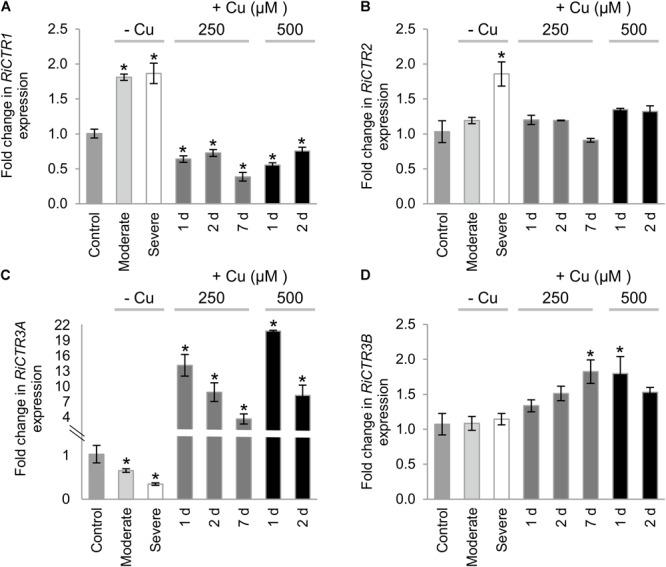
Regulation of *RiCTRs* expression by Cu availability. *R. irregularis* ERM was grown in monoxenic cultures in M media containing 0.5 μM CuSO_4_ (control) or in M media lacking Cu in plates started with roots and inoculum previously grown either in M media containing 0.5 μM CuSO_4_ (moderate Cu deficiency) or in M media without Cu (severe Cu deficiency). For the Cu toxicity treatments, the ERM grown in optimal M media was exposed for 1, 2, and 7 days to 250 μM CuSO_4_ or for 1 and 2 days to 500 μM CuSO_4_. **(A)** *RiCTR1*, **(B)** *RiCTR2*, **(C)** *RiCTR3A* and **(D)** *RiCTR3B* gene expression. Relative expression levels were calculated by the 2^-ΔΔCT^ method using *RiEF1α* as a normalizer. Bars represent standard error. Asterisks show statistically significant differences (*P* < 0.05; *n* = 3) in comparison to the corresponding control value.

*RiCTR1* expression was up-regulated by Cu deficiency and down-regulated by Cu toxicity. A twofold induction was observed in the ERM grown both under moderate and severe Cu limiting conditions (Figure 6A). In contrast, *RiCTR2* transcript levels were significantly increased (twofold) only in the ERM grown under the severe Cu-limiting treatment. *RiCTR2* expression was not affected by any of the toxic Cu conditions considered in our study (Figure 6B).

The expression levels of *RiCTR3A* and *RiCTR3B* were similar in the control untreated ERM. Interestingly, *RiCTR3A* expression was highly up-regulated in ERM subjected to the Cu toxicity treatments and down-regulated in the ERM grown under Cu limiting conditions. *RiCTR3A* induction by Cu toxicity seemed to be transient, reaching a maximum expression level (>20-fold induction) in the ERM exposed to 500 μM CuSO_4_ for 1 day (Figure 6C). This expression pattern was unexpected for a gene encoding a protein that transports Cu into the cytosol and suggests a role for RiCTR3A in Cu tolerance. *RiCTR3B* expression was just slightly up-regulated in the ERM grown for 7 d at 250 μM Cu and for 1 d at 500 μM Cu (Figure 6D). Differential regulation of the two *RiCTR3* splicing variants by Cu leads to higher transcript levels of *RiCTR3A* than of *RiCTR3B* under Cu toxic levels and to higher transcript levels of *RiCTR3B* under the severe Cu deficient treatment (Supplementary Figure S6).

### *RiCTRs* Expression Is Regulated by Oxidative Stress

**FIGURE 7:**
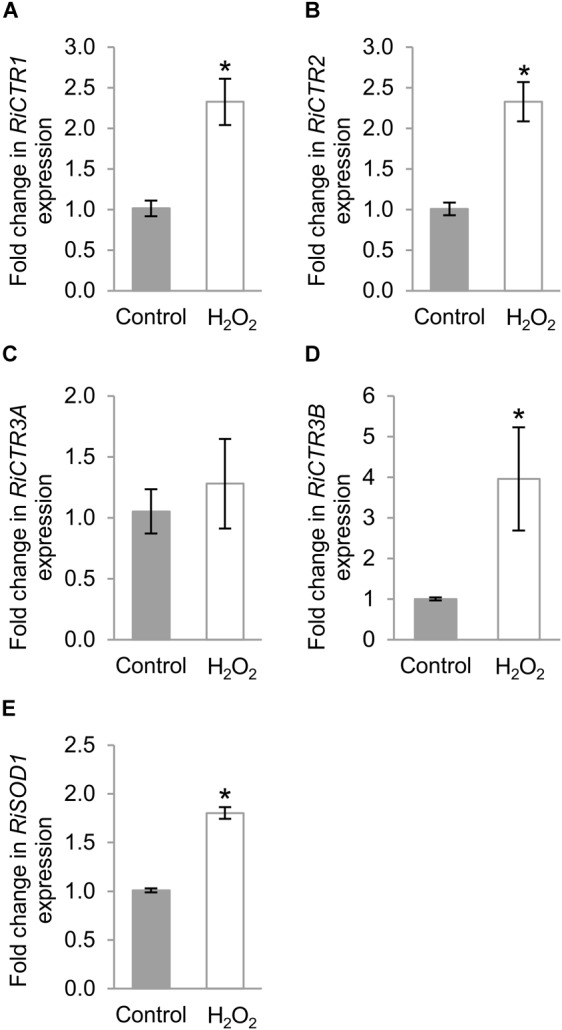
Regulation of *RiCTRs* expression by oxidative stress. *R. irregularis* ERM grown in monoxenic cultures in M-C medium was exposed or not (Control) for 1 h to 1 mM H_2_O_2_. **(A)** *RiCTR1*, **(B)** *RiCTR2*, **(C)** *RiCTR3A* **(D)** *RiCTR3B*, and **(E)** *RiSOD1* gene expression. Relative expression levels were calculated by the 2^-ΔΔCT^ method using *RiEF1α* as a normalizer. Bars represent standard error. Asterisks show statistically significant differences (*P* < 0.05; *n* = 3) in comparison to the control value.

Taking into account that several oxidative-stress response elements were identified in the promoter sequences of the *R. irregularis CTR* genes and that toxic Cu levels induce an oxidative stress to the ERM (Benabdellah et al., 2009), in an attempt to further understand the role of the *R. irregularis* CTRs, their gene expression patterns were analyzed in the ERM exposed to H_2_O_2_. As a marker of the oxidative stress treatment, the expression of the *R. irregularis* Cu, Zn superoxide dismutase gene *RiSOD1* (González-Guerrero et al., 2010) was determined. Exposure of the ERM to 1 mM H_2_O_2_ for 1 h up-regulated *RiCTR1, RiCTR2, RiCTR3B* and *RiSOD1* expression (Figure 7A,B,D,E). However, *RiCTR3A* expression was not significantly affected by H_2_O_2_, which indicates that its activation by Cu was independent of the Cu-induced oxidative stress (Figure 7C). Differential regulation of the two *RiCTR3* splicing variants by H_2_O_2_ leads to higher *RiCTR3B* transcript levels in the H_2_O_2_-treated ERM (Supplementary Figure S7).

### RiCTR3A Enhances Metal Tolerance of the *yap1Δ* Yeast Strain

**FIGURE 8:**
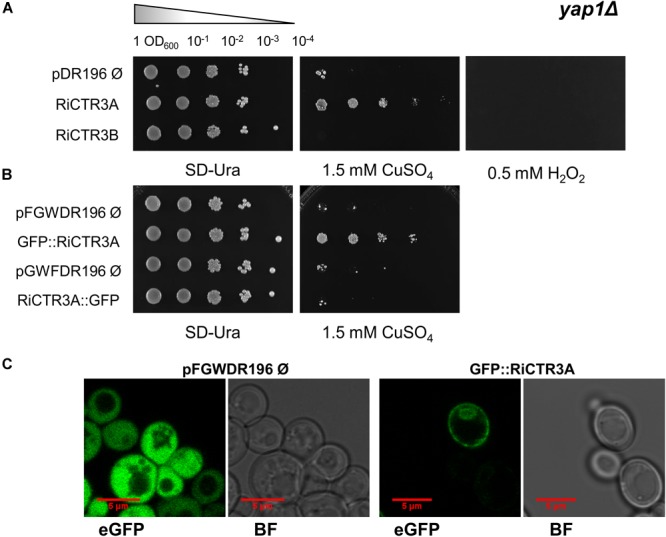
Functional analysis of RiCTR3A and RiCTR3B in the *Δ-yap1 S. cerevisiae* mutant. **(A)** *Δ-yap1* cells transformed with the empty vector or expressing *RiCTR3A* or *RiCTR3B* were plated on SD media supplemented or not with 1.5 mM CuSO_4_ or with 0.5 mM H_2_O_2_. **(B)** *Δ-yap1* cells transformed with the corresponding empty vector expressing *GFP* or N-terminal or C-terminal GFP-tagged versions of *RiCTR3A* were plated on SD media supplemented or not with 1.5 mM CuSO_4_. Plates were incubated 4 days at 30°C. **(C)** *Δ-yap1* cells expressing *GFP* (the empty vector pFGWDR196) and *GFP::RiCTR3A* were visualized with a confocal microscope. eGFP, enhanced GFP fluorescence; BF, bright field.

As a step forward to understand RiCTR3A and RiCTR3B function and taking into account that their transcript levels were regulated by Cu toxicity or H_2_O_2_, we assessed their capability to rescue metal and H_2_O_2_ sensitivity of the *yap1Δ S. cerevisiae* strain lacking the transcriptional regulator Yap1 that mediates cell’s response to oxidants and metals. Neither the empty vector-transformed cells nor the *RiCTR3B*-expressing cells were able to grow on SD media supplemented with Cu or H_2_O_2_ (Figure 8A). However, RiCTR3A rescued the growth defect of the mutant yeast in media supplemented with 1.5 mM CuSO_4_ but not the inability of the *yap1Δ* cells to grow in the presence of 0.5 mM H_2_O_2_ (Figure 8A). These data indicate that RiCTR3A plays, at least in the heterologous system, a role in Cu tolerance. To determine RiCTR3A subcellular location in *yap1Δ*, N- and C-terminal GFP-tagged versions of RiCTR3A were expressed in the mutant yeast cells. However, only GFP-RiCTR3A remained functional (Figure 8B). This fusion protein was localized to the yeast plasma membrane. Additionally, as usually occurs when transport proteins are overexpressed in yeast, a perinuclear fluorescence pattern indicative of endoplasmic reticulum localization was observed (Figure 8C).

## Discussion

The ability of AM fungi to acquire Cu from the soil and to transfer it to their host plants has been shown in several physiological studies. Whereas much progress has been made in understanding the mechanisms of phosphorus and nitrogen transport in the AM symbiosis, very little is known about the mechanisms of Cu acquisition by AM fungi. Here, we characterize for the first time the Cu transporters of the CTR family in an AM fungus. Our data strongly suggest that *R. irregularis* acquires Cu (I) from the soil through the activity of RiCTR1, a plasma membrane Cu transporter that is highly expressed in the ERM, and that RiCTR2 and RiCTR3A play a role in Cu homeostasis in *R. irregularis*.

A previous genome-wide analysis of Cu transporters in *R. irregularis* revealed the presence of three candidate gene sequences, *RiCTR1, RiCTR2*, and *RiCTR3*, encoding transporters belonging to the CTR family (Tamayo et al., 2014). Interestingly, our RACE approach identified two *RiCTR3* transcripts, which result from an alternative splicing event through the retention of the first intron in its coding sequence, the most common alternative splicing type described in fungi (Grutzmann et al., 2014; Gonzalez-Hilarion et al., 2016). Alternative splicing is a common mechanism used to produce multiple proteins from a single gene, thereby increasing the proteome size of an organism (Black, 2003; Benabdellah et al., 2007; Kornblihtt et al., 2013; Mockenhaupt and Makeyev, 2015). In addition, it can influence gene expression through its impact on different stages of mRNA metabolism including transcription, polyadenylation, nuclear mRNA export, translation efficiency and the rate of mRNA decay (Le Hir et al., 2003; Jacob and Smith, 2017). Although functionality of alternative splicing is poorly understood in fungi, it seems that usually leads to non-functional isoforms, providing an additional mechanism to regulate the overall expression of a gene (Goebels et al., 2013; Grutzmann et al., 2014; Gonzalez-Hilarion et al., 2016; Jin et al., 2017; Sieber et al., 2018). However, the extent and biological significance of this process in AM fungi is currently unknown.

CTR proteins contain three TM regions, with a characteristic MetXXXMet motif located in the second TM domain that is absolutely necessary for Cu transport, and an amino-terminal region rich in Met motifs (De Feo et al., 2007). Although most of these methionine motifs are dispensable for Cu transport, a Met/Cys-X-Met motif near the first TM domain is essential for function (Puig et al., 2002). Our *in silico* analyses revealed that out of the four identified RiCTR open reading frames, only RiCTR1 and RiCTR2 present all the structural features of CTR proteins. In fact, these two proteins were the only *R. irregularis* CTRs displaying Cu transport activity in the heterologous system. The finding that RiCTR1 reverts the mutant phenotype of the *ctr1Δctr3Δ* strain lacking the high affinity plasma membrane Cu transporters Ctr1 and Ctr3 indicates that RiCTR1 encodes a plasma membrane Cu transporter that transports Cu (I). Localization of RiCTR1 in the yeast plasma membrane and *RCTR1* expression patterns in the ERM in response to external Cu, that is up-regulation by Cu deficiency and down-regulation by Cu toxicity, supports this hypothesis. Although RiCTR2 subcellular localization could not be demonstrated in the heterologous system, it seems to be the functional ortholog of the *S. cerevisiae* vacuolar Cu transporter Ctr2, as it complemented the growth defect of the triple CTR mutant yeast *ctr1Δctr2Δctr3Δ* lacking both the vacuolar and plasma membrane transporters but not of the double mutant lacking only the plasma membrane transporters. These data strongly suggest a role for RiCTR2 in mobilization of vacuolar Cu stores, which is supported by up-regulation of *RiCTR2* expression when the ERM was grown under the severe Cu deficient conditions. Therefore, as it has been shown for the *S. cerevisiae* plasma membrane transporters Ctr1 and Ctr3 (Dancis et al., 1994a; Peña et al., 2000) and the vacuolar transporter Ctr2 (Rees et al., 2004), RiCTR1 is required to facilitate Cu acquisition under Cu deficient conditions and RiCTR2 to mobilize Cu vacuolar stores when Cu levels are extremely low.

Interestingly, *RiCTR1* and *RiCTR2* transcript levels raised in the ERM in response to H_2_O_2_. A potential explanation could be that under these conditions RiCTR1 and RiCTR2 are needed to increase Cu availability for Cu/Zn-superoxide dismutase, one of the cofactors needed for its reactive oxygen species scavenging activity. In fact, yeast cells lacking CTR transporters show oxidative stress sensitive phenotypes linked with an insufficient delivery of Cu, either from the external environment or from the vacuolar reserves, to the Cu/Zn superoxide dismutase (Dancis et al., 1994a; Knight et al., 1996). These data suggest, therefore, a role for Cu in oxidative stress protection in *R. irregularis*.

As reported for the *R. irregularis* genes *RiPT* (Fiorilli et al., 2013) *RiAMT1-3* (Pérez-Tienda et al., 2011; Calabrese et al., 2016) and *RiFTR1* (Tamayo et al., 2018) encoding, respectively, plasma membrane phosphate, ammonium and iron transporters, *RiCTR1* and *RiCTR2* mRNAs were detected in the IRM. Expression of *RiCTR1* in the IRM suggests, as it has been proposed for the other fungal transporters, that there might exist a competition between the plant and the fungus for the Cu present in the apoplast of the symbiotic interface (Balestrini et al., 2007; Kiers et al., 2011). It is tempting to hypothesize that during its *in planta* phase, the fungus needs to take up Cu from the interfacial apoplast to meet its Cu demands for growth and activity. This hypothesis is supported by the observed increase of the *RiCTR1* transcript levels in the IRM when the symbiosis was developed under Cu-limiting conditions. Under these conditions, Cu released by the fungus into the apoplast of the arbuscular interface should be perceived not only by the plant but also by the fungus. Further experiments are needed to understand how Cu homeostasis is regulated at the symbiotic interface, a process that will require fine-tuning between the plant and the fungus and that will depend on the Cu status of both symbionts. The high expression levels of *RiCTR2* in the IRM together with its up-regulation when the symbiosis was developed under Cu-deficient conditions suggest that the fungus needs to mobilize its vacuolar Cu reserves to support its growth and metabolism. Overall, these data indicate that the fungus has a high Cu demand for growth and activity within the roots, which is supported by the observation that root colonization and arbuscule development are inhibited when the symbiosis was developed under Cu-deficient conditions. The requirement of Cu for AM fungal colonization it is not surprising given that this transition metal is an essential micronutrient that acts as cofactor of key enzymes involved in a wide array of biochemical processes essential for growth (Pena et al., 1999; Festa and Thiele, 2011).

Unlike RiCTR1 and RiCTR2, none of the *RiCTR3* gene products seem to have a role in Cu transport. RiCTR3A presents the typical topology of CTR proteins but lacks the conserved Met/Cys-X-Met motif near the first TM domain that is strictly required for Cu transport; and RiCTR3B has a single TM domain. As expected, neither RiCTR3A nor RiCTR3B restored the respiratory defect of the CTR mutant yeasts. Furthermore, their gene expression patterns in response to external Cu presented the opposite trend of a protein that mediates Cu transport into the cytosol, as both were transiently up-regulated when the ERM was exposed to high Cu levels. The strong up-regulation of *RiCTR3A* expression in the Cu-treated ERM together with the capability of its gene product to revert metal sensitivity of the *Δyap-1* yeast cells suggest that RiCTR3A is involved in metal tolerance in the ERM. Given that RiCTR3A was unable to complement oxidative stress sensitivity of the *Δyap-1* mutant and that a functional GFP-RiCTR3A fusion protein was localized to the *Δyap-1* plasma membrane, it is temping to hypothesize that RiCTR3A might function as a Cu sensor that activates downstream signal transduction pathways involved in Cu tolerance. Nutrient sensing in fungi is mediated by different classes of plasma membrane proteins that activate downstream signaling pathways, such as non-transporting receptors, transceptors and G-proteins-coupled receptors (Van Dijck et al., 2017). Non-transporting receptors are structural homologs to nutrient transporters that have lost their transport capacity while gaining a receptor function (Conrad et al., 2014). It is believed that these transporter-like proteins are used as sensors for the nutrient they likely once transported previously in evolution. This is the case of the *S. cerevisiae* glucose receptors Snf3 and Rgt2, structural homologs glucose transporters that sense availability of external glucose but cannot transport glucose (Ozcan et al., 1998), and of the nitrogen receptor SSy1, a member of the amino acid permease family that does not transport amino acids but senses them at the plasma membrane (Klasson et al., 1999). Despite failure of RiCTR3A to complement the mutant phenotype of the yeast CTR mutants could be an artifact of the heterologous system, the absence in its N-terminal end of the Met/Cys-X-Met motif that is strictly required for CTR function supports the hypothesis that RiCTR3A does not have Cu transport activity. Although micronutrient receptors have been not reported yet, the yeast iron transporter Ftr1 and the zinc transporter Ztr1 have recently been identified as the first micronutrient transceptors, since they present both transport and receptor functions (Schothorst et al., 2017). RiCTR3A might be the first described micronutrient receptor. However, further studies are required to confirm this hypothesis.

Unfortunately, we could not assign a role to *RiCTR3B*, the intron-retaining transcript of *RiCTR3*. Alternative splicing variants of CTR genes have been previously described in other fungi, such as *C. gloeosporioides* (Barhoom et al., 2008) and *N. crassa* (Korripally et al., 2010). However, in contrast with what happens with the protein encoded by *RiCTR3B*, the predicted proteins of the two spliced variants of the *C. gloeosporioides CTR2* gene and of the *N. crassa* TCU-2 present all the characteristic features of CTR proteins and their gene products are fully functional in the yeast Ctr triple mutant. Since intron retention in *RiCTR3B* produces a frame shift that generates a premature termination codon, it is possible that the alternative *RiCTR3* protein RiCTR3B is not produced. If that were the case, as it has been described for other fungi (Gonzalez-Hilarion et al., 2016), intron retention might be a post-transcriptional mechanism to regulate *RiCTR3* gene expression. A systemic genome-wide comparative analysis of alternative splicing in 23 fungal species has revealed that most of the alternative splicing-affected genes encode proteins that mediate the stress response (Grutzmann et al., 2014). Interestingly, RiCTR3A seems to be involved in the ERM response to Cu toxicity. The finding that both *RiCTR3* splicing variants were differentially expressed during Cu and oxidative stress agrees with previous observations in several human pathogenic fungi that the expression of a certain isoform is not exclusive to a certain condition and that the ratio between expressed isoforms changes (Sieber et al., 2018). These authors suggested that alternative splicing is important in fungi for adaptation and stress tolerance via the generation of suitable splice variants. The higher expression levels of the *RiCTR3A*, the transcript lacking the first intron, under Cu toxicity suggests that alternative splicing may be a mechanism to control the activation of the RiCTR3A protein during Cu stress. The finding that *RiCTR3B* expression increased in the H_2_O_2_-exposed ERM suggests that it might play a role in oxidative stress tolerance. However, further studies are needed to determine whether *RiCTR3B* encodes a functional protein and the significance of the alternative splicing of *RiCTR3*.

## Conclusion

Here, we show for the first time that the AM fungus *R. irregularis* expresses two genes encoding Cu transporters of the CTR family, *RiCTR1* and *RiCTR2*, and two alternative spliced variants of a third gene, *RiCTR3*. *RiCTR3A*, the shortest spliced variant of *RiCTR3*, encodes a protein that is likely involved in Cu tolerance while *RiCTR3B* might contribute to oxidative stress protection. Our data also show for the first time the requirement of Cu for AM fungal colonization.

## Data Availability

The raw data supporting the conclusions of this manuscript will be made available by the authors, without undue reservation, to any qualified researcher.

## Author Contributions

TG-G performed the majority of the experimental work. KB, MM, and AJ-J contributed to RiCTR2 characterization. CA and PB contributed to the protein localization assays. NF defined the research theme, supervised all the experiments, and coordinated the research project. TG and NF contributed to data interpretations and wrote the manuscript. All authors have revised and approved the manuscript.

## Conflict of Interest Statement

The authors declare that the research was conducted in the absence of any commercial or financial relationships that could be construed as a potential conflict of interest.

## Footnotes

1 https://www2.dijon.inra.fr/mychintec/Mycocalc-prg/download.html

2 https://genome.jgi.doe.gov/portal/

3 https://blast.ncbi.nlm.nih.gov/Blast.cgi

4 https://www.ebi.ac.uk/Tools/msa/clustalo/

5 http://rulai.cshl.edu/SCPD/

6 http://rsat-tagc.univ-mrs.fr/rsat/

7 http://www.bioinformatics.org/sms2/ident_sim.html

8 https://www.ncbi.nlm.nih.gov/Structure/cdd/wrpsb.cgi

9 http://www.cbs.dtu.dk/services/TMHMM/

10 http://harrier.nagahama-i-bio.ac.jp/sosui/sosui_submit.html

11 http://topcons.cbr.su.se/

12 https://prosite.expasy.org/mydomains/
