# Supplementary material for: The Rhizophagus irregularis Genome Encodes Two CTR Copper Transporters That Mediate Cu Import Into the Cytosol and a CTR-Like Protein Likely Involved in Copper Tolerance
Source: Front Plant Sci. 2019 May 16;10:604. doi: 10.3389/fpls.2019.00604 (PMC6531763; doi:10.3389/fpls.2019.00604)
Supplement: Supplementary file 1 [file Data_Sheet_1.pdf]

Supplementary Table 1. ***Saccharomyces cerevisiae*** **strains used in this work.**

Strain	Genotype	Reference
**MPY17**	*MATα ctr1::ura3::Kan^r ctr3::TRP1 his3 lys2-801 CUP1^R*	(Peña et al., 1998)
**MPY17 *ctr2Δ***	*MATα ctr1::ura3::Kan^r ctr3::TRP1 ctr2::HIS3 lys2-801 CUP1^R*	(Rees et al., 2004)
**WYT**	*MATα his3 can1-100 ade2 leu2 trp1 ura3 yap1:: TRP1*	(Kuge and Jones, 1994)

Supplementary Table 2. Primers used in experimental procedures. Overlaps with the corresponding cloning vectors and restriction sites are highlighted in bold or underlined, respectively. w/o: without

Primer ID	Sequence (5'-3')	Purpose
RiCTR1R.5r	ATCAATTGTCCTGTAGTCAAGCCCGTC	5'RACE of *RiCTR1*
RiCTR2R.5r	CATGCATCGCTATCATGACTACCACCATG	5'RACE of *RiCTR2*
RiCTR3R.5ra	AGTATGAAATGAGGTAACTTGGAACCCT	5'RACE of *RiCTR3*
RiCTR3R.5rb	CCCTGCATAAGTAAATGTCCTTATTACAACC	5'RACE of *RiCTR3*
RiCTR3R.5rc	**GATTACGCCAAGCTT**GGAGATCATCATCTTGCGAAATTGGAAC	5'RACE of *RiCTR3*
RiCTR1F.3r	ACAGAACATACAACCCCAGCTCACTTCG	3'RACE of *RiCTR1*
RiCTR3F.3r	CAGGGTTCCAAGTTACCTCATTTCATACT	3'RACE of *RiCTR3*
RiCTR1FL.F	TCCCCCGGGATGTCAGAGACAATGACAGAAC	Cloning of *RiCTR1* CDS into pDR196
RiCTR1FL.R	CCGCCTCGAGTTATGTCCTCTTTTCCTCCTTG	Cloning of *RiCTR1* CDS into pDR196
RiCTR2FL.F	AACTGCAGATGAATTATCAACAATTT	Cloning of *RiCTR2* CDS into pDR196
RiCTR2FL.R	ACGCGTCGACTTAATGGCAAGTTATTGA	Cloning of *RiCTR2* CDS into pDR196
RiCTR3AFL.F	AACTGCAGATGCTTTATTTTACGAGTGATTTG	Cloning of *RiCTR3A* CDS into pDR196
RiCTR3BFL.F	AACTGCAGATGCTATTAATAATGTCGATGAATTC	Cloning of *RiCTR3B* CDS into pDR196
RiCTR3FL.R	ACGCGTCGACTCATTTATTAACGAGACTTTGCTG	Cloning of *RiCTR3A/B* CDS into pDR196
RiCTR3FLg-F	ATATACGCAGCCTCATAAATTC	*RiCTR3* genomic clone
RiCTR1gwF	**CACC**ATGTCAGAGACAATGACAGAAC	Cloning of *RiCTR1* CDS into pENTR/D-TOPO
RiCTR1gwR	TTATGTCCTCTTTTCCTCCTTG	Cloning of *RiCTR1* CDS into pENTR/D-TOPO
RiCTR1gwNoSTOP-R	TGTCCTCTTTTCCTCCTTG	Cloning of *RiCTR1* CDS w/o stop codon into pENTR/D-TOPO
RiCTR2gwF	**CACC**ATGAATTATCAACAATTTATCATAAAAG	Cloning of *RiCTR2* CDS into pENTR/D-TOPO
RiCTR2gwR	TTAATGGCAAGTTATTGATTTACC	Cloning of *RiCTR2* CDS into pENTR/D-TOPO
RiCTR2gwNoSTOP-R	ATGGCAAGTTATTGATTTACC	Cloning of *RiCTR2* CDS w/o stop codon into pENTR/D-TOPO
RiCTR3AgwF	**CACC**ATGCTTTATTTTACGAGTGATTTG	Cloning of *RiCTR3A* CDS into pENTR/D-TOPO

Primer ID	Sequence (5'-3')	Purpose
RiCTR3AgwR	TCATTTATTAACGAGACTTTGCTG	Cloning of *RiCTR3A* CDS into pENTR/D-TOPO
RiCTR3AgwNoSTOP-R	TTTATTAACGAGACTTTGCTGC	Cloning of *RiCTR3A* CDS w/o stop codon into pENTR/D-TOPO
RICTR1Fq	TATAGTGGTGACGGGCTTGACT	Real-Time qPCR of *RiCTR1*
RICTR1Rq	GAAGGGTGAATTGTATATTGTTGG	Real-Time qPCR of *RiCTR1*
RiCTR2Fq	GGTGGTAGTCATGATAGCGATG	Real-Time qPCR of *RiCTR2*
RiCTR2Rq	CTATTTACGCGCCACCAAGT	Real-Time qPCR of *RiCTR2*
RiCTR3AFq	GCTTTATTTTACGAGTGATTTG	Real-Time qPCR of *RiCTR3A*
RiCTR3ARq	TTGTCAATCCAGTTACCACCAC	Real-Time qPCR of *RiCTR3A*
RiCTR3BFq	TGGTAAAATATTTCATTAGTTACTCTGAC	Real-Time qPCR of *RiCTR3B*
RiCTR3BRq	ATCTCAAAATTGTTAACCCTGCAT	Real-Time qPCR of *RiCTR3B*
RiMST2Fq	GGCAGGATATTTGTCTGATAG	Real-Time qPCR of *RiMST2*
RiMST2Rq	GCAATAACTCTTCCCGTATAC	Real-Time qPCR of *RiMST2*
RiSOD1Fq	GTACTATTACTTTCATTCAGGA	Real-Time qPCR of *RiSOD1*
RiSOD1Rq	AGTTCATGACCACCTTTACCAA	Real-Time qPCR of *RiSOD1*
RiEF1αFq	GCTATTTTGATCATTGCCGCC	Real-Time qPCR of *RiEF1α*
RiEF1αRq	TCATTAAAACGTTCTTCCGACC	Real-Time qPCR of *RiEF1α*
CiEF1αFq	CATGCGTCAGACGGTTGCTGT	Real-Time qPCR of *CiEF1α*
CiEF1αRq	CTTCACTCCCTTCTTGGCTGC	Real-Time qPCR of *CiEF1α*
DcEF1αFq	TCAAGGATCTCAAGCGTGGTTATGT	Real-Time qPCR of *DcEF1α*
DcEF1αRq	CAGCAATGTGGCAAGTGTGACAAT	Real-Time qPCR of *DcEF1α*

Supplementary Table 3. Comparison among the predicted amino acid sequence of the *R. irregularis* CTR family members. Percentages of identity/similarity are indicated.

	**RiCTR1**	**RiCTR2**	**RiCTR3A**	**RiCTR3B**
RiCTR1	100/100	16/24	39/53	21/27
RiCTR2		100/100	11/20	4/9
RiCTR3A			100/100	55/55
RiCTR3B				100/100

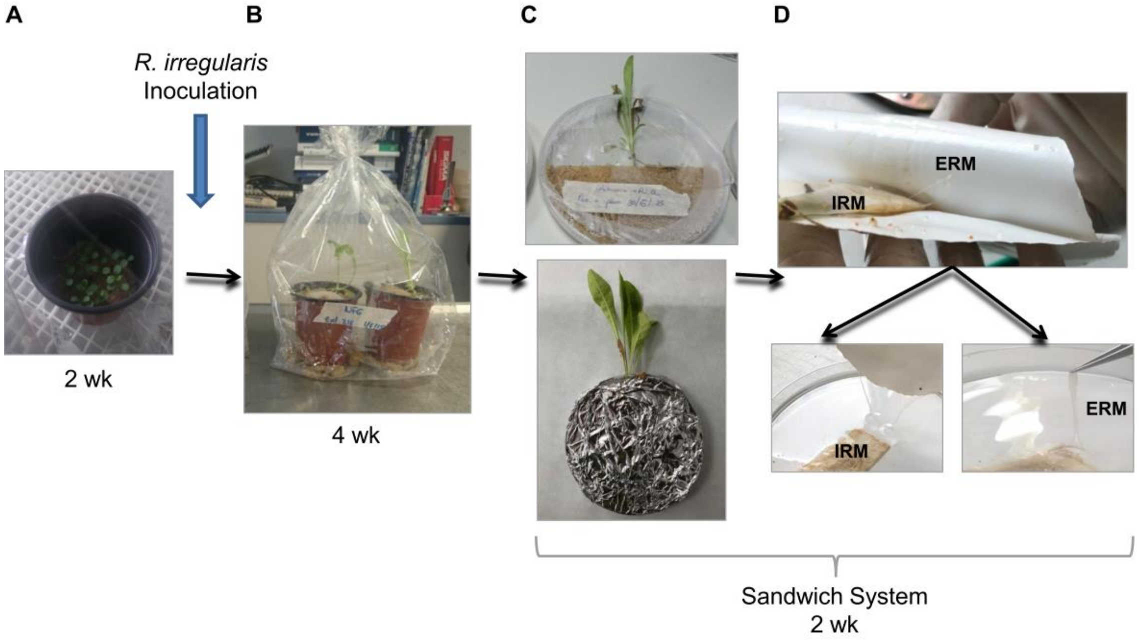

Supplementary Figure 1. Scheme of the *in vivo* whole plant bidimensional experimental system described by Pepe et al. (2017), with some modifications. (**A**) Surface-sterilized chicory (*Cichorium intybus* L.) seeds were germinated for two weeks. (**B**) Seedlings were inoculated with spores, ERM and colonized roots obtained from *R. irregularis* monoxenic cultures and transplanted into 50 mL pots filled with sterile sand. Pots were placed in sun-transparent bags and maintained during one month in a growth chamber (24ºC/21ºC day/night, 16 h light photoperiod). (**C**) A sandwich culture system was established as detailed in Materials and Methods and maintained during two weeks in a growth chamber under the conditions described above. (**D**) ERM spreading from the nylon net onto the membranes was collected with tweezers, while mycorrhizal roots were wrapped in the nylon net.

```
RiCTR1      ------------------------------------------------------------    0
RiCTR3A     ------------------------------------------------------------    0
RiCTR3B     ------------------------------------------------------------    0
ScCTR1      MEGMNMGSSMNMDAMSSASKTVASSMASMSMDAMSSASKTILSSMSSMSMEAMSSASKTL    60
ScCTR3      ------------------------------------------------------------    0
ScCTR2      ------------------------------MDDKKTWSTVTLR-----------------    13
RiCTR2      ------------------------------------------------------------    0

RiCTR1      --------------------------------------------MSE-------TMTEHT    9
RiCTR3A     ------------------------------------------------------------    0
RiCTR3B     ------------------------------------------------------------    0
ScCTR1      ASTMSSMASMSMGSSSMSGMSMSMSSTPTSSASAQTTSDSSMSGMSGMSSSDNSSSSGM-    119
ScCTR3      ------------------------------------------MNMGG-------SSSTAA    11
ScCTR2      --TFNQ-----LVTSSLIG--------------YSKKMDSMNHKMEGNAGHDHSDMHMGD    52
RiCTR2      -----------------MN--------------YQQFI---IKAM----NYD---HGGSH    19

                                              TM1
RiCTR1      TPAHFDHNKL---YFSDDLSKISI----LQVNSIFS--------FVLASIF--V----TL    48
RiCTR3A     --------ML---YFTSDLSKVVILFSGFQVTSFHT--------FVLATLF--V----IS    35
RiCTR3B     ------------------------------------------------------------    0
ScCTR1      ---DMDMSMGMNYYLTPTYKNYPVLFHHLHANNSG----KAFGIFLLFVVAAFVYKLLLF    172
ScCTR3      KKATCKISMLWNWYTI----DTCFIARSWRNDTKGKFAGSCIGCFALVVVAQWL----TR    63
ScCTR2      GDDTCSMNMLFSWSYK----NTCVVFEWWHIKTLPGLILSCLAIFGLAYLYEYL----KY    104
RiCTR2      DSDACNMQMLFNWDTE----KLCIIFTWWRVNSIGFLILSCFIIILLATSFEFL----RF    71

RiCTR1      ICWCERFLN---------YYYHK-----KSK-----------------------------    65
RiCTR3A     LCWCERLFT---------YYYEN------AG-----------------------------    51
RiCTR3B     ------------------------------------------------------------    0
ScCTR1      VSWCLEVHWFKKWDKQNKYSTLPSANSKDEGKHYDTENNFEIQGLPKLPNLLSDIF----    228
ScCTR3      FSRQFDVELLKRQKIKHLASYSPEEYVVKCGEEDAKSDIEELQGFYNEPSWKTTLISLQK    123
ScCTR2      CVHKRQLSQ--------RVLLPN-------------------------------------    119
RiCTR2      ALRKYDTKI--------QVSYSQLQ----EGRD-AN--SEEVA-----------------    99
                                                          TM2
RiCTR1      ---------------------------------YDRNQKRFRSIAVKTLSYGILTTLRLS    92
RiCTR3A     ---------------------------------YEKGQKRFKRVVIRTFTYAGLTILRFF    78
RiCTR3B     ------------------------------------------------------------    0
ScCTR1      --------------------------------VPS--LMDLFHDIIRAFLVFTSTMIIYM    254
ScCTR3      SFIYSFYVWGPRRLNEPEDDLLKKVLSCCTLITPVDLYPTFLDHMIRVTIFVLQWGLSYI    183
ScCTR2      ---------------------------------RSLTKINQADKVSNSILYGLQVGFSFM    146
RiCTR2      ---------------------T----------QSNANRITYVQQLVRTLIHTSQVFLSFF    128

                              TM3
RiCTR1      YMLLIMSMNSQIFIIVVTGLTTGQLIVEYIRSIPIYQQYTIHPSSEITPNTIEGSVLMGN    152
RiCTR3A     YMLLIMSMNSQIFIVVVTGLTTGQLIVEYVCSISVYSTHQLVPISQDD-------DLPTL    131
RiCTR3B     -MLLIMSMNSQIFIVVVTGLTTGQLIVEYVCSISVYSTHQLVPISQDD-------DLPTL    52
ScCTR1      LMLATMSFVLTYVFAVITGLALSEVFFNRCKIAMLKRWD-------IQREIQKAKSCPGF    307
ScCTR3      IMLLFMYYNGYIIISCLIGAIVGRFIFCYEPLGSLGANGSAQGTVSYDKESDDRKCCL--    241
ScCTR2      LMLVFMTYNGWLMLAVVCGAIWGNYSWCTSYSPEIDDSSLACH-----------------    189
RiCTR2      LMLVFMTYNGFLMISVIIGAAIGYFVFSSSV-IDFGGKSITCH-----------------    170
             **  *      .:  : *   .           .

RiCTR1      NNNY-RDEIELESRNFVIENINNETKEE--------------------------------    179
RiCTR3A     ANKL-DDELELQPQSLIIENVNSDDEYD--------------------------------    158
RiCTR3B     ANKL-DDELELQPQSLIIENVNSDDEYD--------------------------------    79
ScCTR1      GNCQCGRHPEPSPDPIAVADTTSGSDQSTRLEKNNESKVAISENNQKKTPTQEEGCNCAT    367
ScCTR3      ------------------------------------------------------------    241
ScCTR2      ------------------------------------------------------------    189
RiCTR2      ------------------------------------------------------------    170

RiCTR1      ------KRT------------------------------       182
RiCTR3A     ------YQIHNDIVKYE----QQSLVNK-----------       176
RiCTR3B     ------YQIHNDIVKYE----QQSLVNK-----------       97
ScCTR1      DSGKNQANIERDILENSKLQEQSGNMDQNLLPAEKFTHN       406
ScCTR3      ---------------------------------------       241
ScCTR2      ---------------------------------------       189
RiCTR2      ---------------------------------------       170
```

Supplementary Figure 2. Alignment of the deduced amino acid sequences of the *R. irregularis* CTRs with the amino acid sequences of the *S. cerevisiae* CTR proteins. Conserved features of this family are highlighted in different colors. In blue: Met residues located in the N terminal region, in red the Met and Gly residues included in the MetXXXMet-X$_{12}$-GlyXXXGly signature, in green: Cys and His residues located in the C terminus and in gray: predicted transmembrane domains (TM).

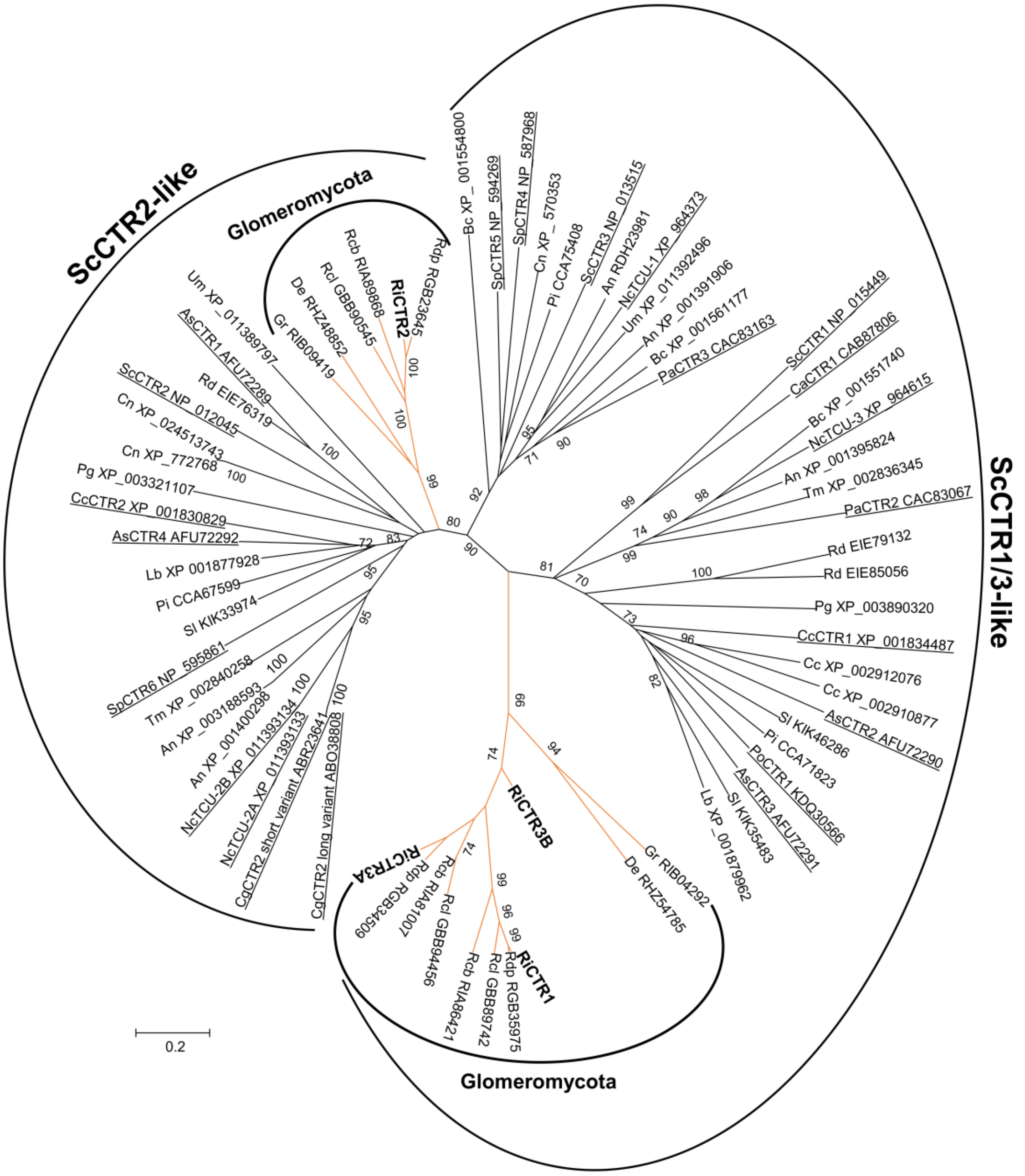

Supplementary Figure 3. Phylogenetic relationships between fungal CTR proteins. The unrooted Neighbor-joining tree was generated using MEGA v. 6 with 1,000 bootstrap replicates. *R.irregularis* CTR proteins are in bold. Functionally characterized CTRs are underlined. GenBank Accession numbers are provided. Organisms: As, *Amanita strobiliformis*; Bc, *Botrytis cinerea*; Ca, *Candida albicans*; Cc, *Coprinopsis cinerea*; Cg, *Colletotrichum gloeosporioides*; Cn, *Cryptococcus neoformans*; De, *Diversispora epigaea*; Gr, *Gigaspora rosea*; Lb, *Laccaria bicolor*; Nc, *Neurospora crassa*; Pa, *Podospora anserina*; Pi, *Piriformospora indica*;Pg, *Puccinia graminis*; Po, *Pleurotus ostreatus*; Sc, *Saccharomyces cerevisiae*; Sl, *Suillus luteus*; Sp, *Schizosaccharomyces pombe*; Tb, *Tuber melanosporum*; Rcb, *Rhizophagus cerebriforme*; Rcl, *Rhizophagus clarus*; Rdp, *Rhizophagus diaphanous*; Ri, *Rhizophagus irregularis*; Rd, *Rhizopus delemar*; Um, *Ustilago maydis*. Bootstrap values above 70 and supporting a node used to define a cluster are indicate.

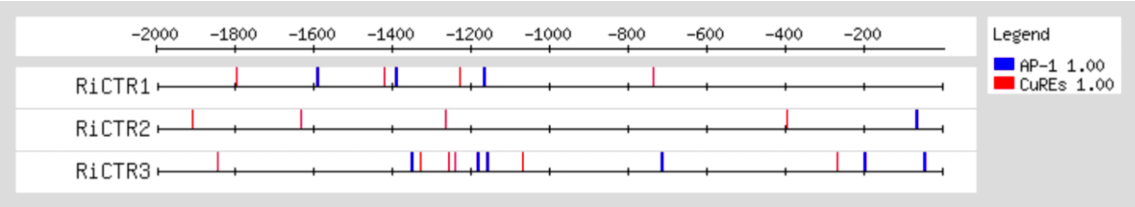

Supplementary Figure 4. Putative *cis*-regulatory elements in the promoter region of *Rhizophagus irregularis CTRs* genes. *Cis*-regulatory elements were screened in the *Saccharomyces cerevisiae* Promoter Database SCPD (http://rulai.cshl.edu/SCPD/) and specific Cu responsive elements (CuREs) and AP-1 sites were further screened through DNA pattern matching analyses in the fungal RSAT server without allowing any nucleotide substitution (http://rsat-tagc.univ-mrs.fr/rsat/).

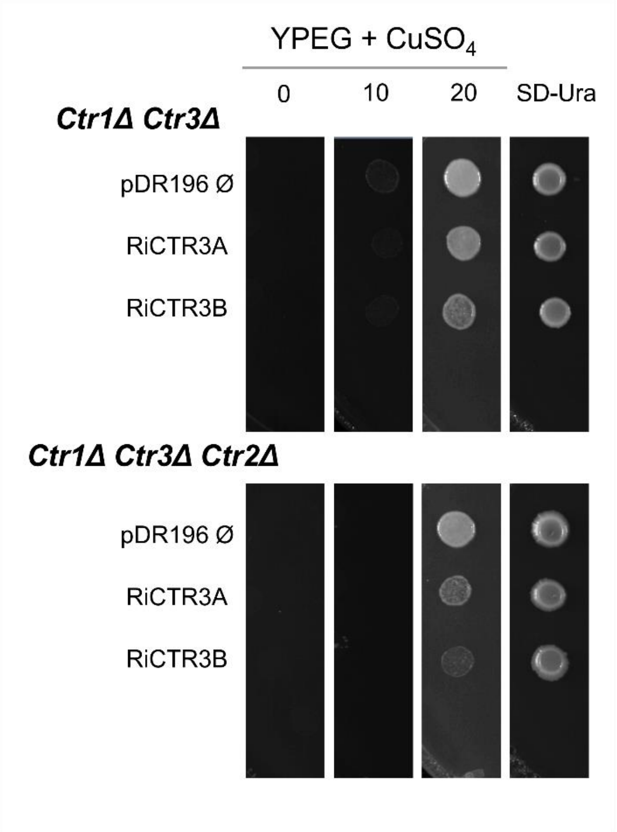

Supplementary Figure 5. Analysis of functionality of RiCTR3 splicing variants in *Saccharomyces cerevisiae* CTR mutants. *ctr1Δctr3Δ* and *ctr1Δctr2Δctr3Δ* yeast cells transformed with the empty vector or expressing *RiCTR3A* or *RiCTR3B* were plated on YPEG media supplemented with Cu (0, 10 or 20 μM $CuSO_4$) or on SD medium without uracil. *ctr1Δctr3Δ* and *ctr1Δctr2Δctr3Δ* plated cells were incubated at 30 °C for 4 and 7 days, respectively.

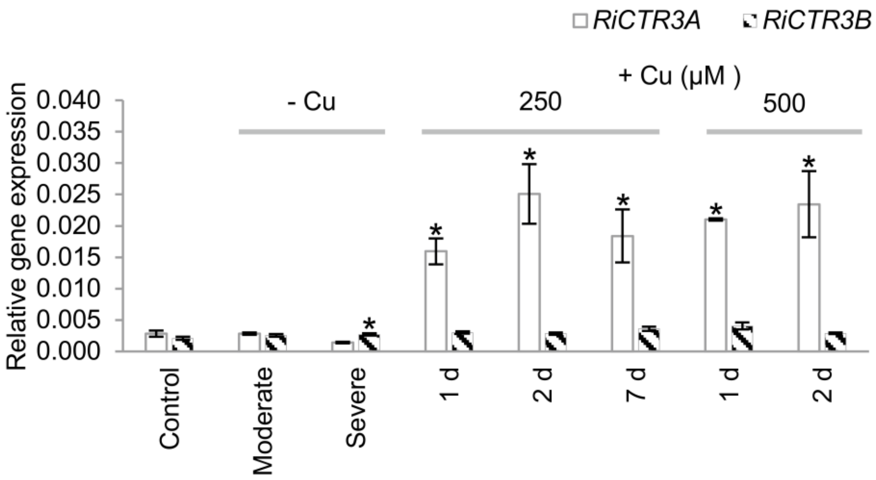

Supplementary Figure 6. Regulation of *RiCTR3A* and *RiCTR3B* expression by Cu availability. *R. irregularis* ERM was grown in monoxenic cultures in M media containing 0.5 µM $CuSO_4$ (control) or in M media lacking Cu in plates started with roots and inoculum previously grown either in M media containing 0.5 µM $CuSO_4$ (moderate Cu deficiency) or in M media without Cu (severe Cu deficiency). For the Cu toxicity treatments, the ERM grown in optimal M media was exposed for 1, 2 and 7 days to 250 µM $CuSO_4$ or for 1 and 2 days to 500 µM CuSO4. Relative expression levels were calculated by the $2^{-\Delta CT}$ method using *RiEF1α* as a normalizer. Bars represent standard error. Asterisks show statistically significant differences ($P < 0.05$; n= 3) in comparison to the corresponding control value.

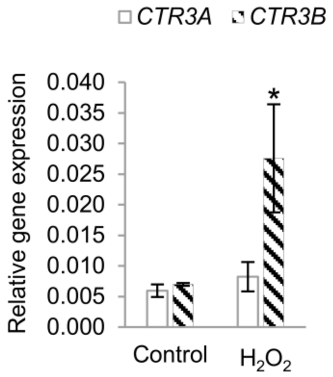

Supplementary Figure 7. Regulation of *RiCTR3A* and *RiCTR3B* expression by oxidative stress. *R. irregularis* ERM grown in monoxenic cultures in M-C medium was exposed or not (control) for 1h to 1 mM H_2O_2. Relative expression levels were calculated by the $2^{-\Delta CT}$ method using *RiEF1α* as a normalizer. Bars represent standard error. Asterisks show statistically significant differences ($P < 0.05$; $n = 3$) in comparison to the corresponding control value.
